# Folate Receptor Alpha (FRα) and the Developing Brain: From Molecular Function to Neurodevelopmental Outcomes

**DOI:** 10.1007/s12035-026-05813-z

**Published:** 2026-03-25

**Authors:** Olga Egorova, Erik Domellöf, Maryam Ardalan, Carina Mallard

**Affiliations:** 1 Department of Clinical Sciences, Pediatrics, Umeå University, Umeå, Sweden; 2 Department of Cognitive Medicine, Ängelholm Hospital, Ängelholm, Sweden; 3 Department of Psychology, Umeå University, Umeå, Sweden; 4 Department of Physiology, Institute of Neuroscience and Physiology, Sahlgrenska Academy, University of Gothenburg, Gothenburg, Sweden; 5 Department of Clinical Medicine, Translational Neuropsychiatry Unit, Aarhus University, Aarhus, Denmark

**Keywords:** Folate receptor alpha, Folate transport, Neurodevelopment, Brain

## Abstract

Folate receptor alpha (FRα) is a high-affinity transporter responsible for delivering folate (vitamin B9) into the central nervous system (CNS), supporting the synthesis of nucleic acids, amino acids, lipids, and signaling molecules through one-carbon transfer pathways. Beyond its transport function, upon activation by folate, FRα modulates extracellular signaling, regulates membrane and cytosolic proteins, and gene transcription. Disruption of FRα, whether functional or structural, can impair CNS folate availability and lead to developmental abnormalities or neurological dysfunction, with the clinical consequences depending on the timing, severity, and presumably on the individual genetic background. Together, these insights position FRα as both a multifunctional regulator of cellular fate and a key molecular interface connecting folate metabolism, brain development, and neurobehavioral outcomes. This narrative review synthesizes the current state of knowledge on FRα, highlighting its roles in neuronal and glial differentiation, CNS maturation, and neural plasticity, while also examining the links between impaired FRα function and a spectrum of developmental and neuropsychiatric conditions. It also outlines the need for broader population-based studies to evaluate the diagnostic value of FRα autoantibodies and the therapeutic potential of folate supplementation.

## Introduction

Folate (vitamin B9) encompasses a group of structurally related molecules containing a pteridine ring and p-aminobenzoic acid, and glutamate or polyglutamate. These molecules are vital cofactors in numerous cellular processes, including DNA synthesis, repair, methylation, and amino acid metabolism. However, due to their anionic and hydrophilic nature, folates cannot efficiently cross cell membranes by passive diffusion, necessitating specialized transport mechanisms. An adequate folate supply is particularly important for supporting rapidly proliferating or reorganizing tissues such as the developing brain, where demands for nucleotide biosynthesis and epigenetic regulation are high [1, 2].

To overcome cellular impermeability to folate, vertebrates rely on multiple high-affinity transport systems: the reduced folate carrier (RFC), the proton-coupled folate transporter (PCFT), and folate receptors (FRs) [3–6]. These systems function under distinct conditions and are adapted to different folate concentrations. While RFC and FRs facilitate folate uptake at physiological pH (~7.4), PCFT is most active at acidic pH (~5.6) [2, 5] allowing, among other functions, transport from acidified intracellular vesicles. All these transporters are present in the central nervous system (CNS) with distinct roles and expression patterns [7–9].

Among the above-mentioned transport systems, folate receptor alpha (FRα) assumes a pivotal function in regulating folate entry into the CNS, mediating high-affinity uptake and endocytosis mainly at the choroid plexus [10]. Beyond simple folate transport, FRα actively participates in essential neurodevelopmental processes including neurulation, neuronal proliferation, differentiation, myelination, and synaptic plasticity [11–16]. Its involvement extends from early embryogenesis through to postnatal brain maturation and continues to influence cognitive and neurological function throughout life [14, 16, 17]. Importantly, recent studies indicate that FRα functions not only as a transporter but also as a regulator of intracellular signaling pathways, and as a transcription factor modulating gene expression relevant to neural stem cell maintenance and lineage commitment [18–20].

This review highlights FRα’s roles in cellular signaling, discusses its potential role in stabilizing developmental mechanisms. We also emphasize the importance of FRα trafficking and regulation mechanisms, as well as the consequences of its dysfunction. Disruptions in FRα-mediated folate delivery are implicated in severe neurodevelopmental disorders including cerebral folate deficiency syndromes and subsets of autism spectrum disorder (ASD) [21, 22], indicating the need for enhanced understanding of FRα biology to inform therapeutic strategies.

## Folate Receptor Alpha: Biochemical Properties, Molecular Structure, Localization and Transport

FRα, the focus of this review, is a member of the folate receptor family along with three other folate receptors: FRβ, FRγ, and FRδ [23]; however, only FRα and FRβ are observed in human and rodent CNS tissue [23, 24].

Human folate receptor alpha mRNA encodes 257 amino acids, corresponding to a protein precursor with a molecular weight of almost 30 kDa. The mature core protein of the folate receptor has a molecular weight of 26 kDa [25]. Posttranslational modifications, including N-glycosylation at three sites and binding to a GPI anchor, increase its mass to 38–42 kDa. GPI anchoring secures membrane localization, and glycosylation ensures the receptor’s folate-binding capacity. While FRα is glycosylated on three sites, FRβ has only two sites for N-glycosylation [26–30].

The FRα receptor binds folate in a 1:1 ratio with exceptionally high affinity (dissociation constant less than 10⁻⁹M) at physiological pH (7.4), making it the highest-affinity folate transporter known. In comparison, FRβ has more than ten times lower affinity to physiologically active folate species [5]. This high binding capacity allows FRα to transport folate against a concentration gradient at levels as low as 0.1 nM [5, 26], roughly ten times lower than required transport via RFC [2, 3], making it the most efficient plasma membrane folate transporter identified.

An essential feature of FRα is its higher affinity for folic acid, the oxidized and most stable form of folate, than for 5-methyltetrahydrofolate (5-MTHF), the main circulating folate [31]. This preferential binding has important implications in the context of wheat and maize flour fortification programs using folic acid as the fortificant. Resulting instantaneous intake levels may exceed ~200 µg and lead to the appearance of unmetabolized folic acid (UMFA) in the circulation, including in cord blood [32]. Although folic acid fortification has been highly effective in reducing neural tube defects, accumulating evidence and recent reviews have raised concerns that chronic UMFA exposure may have unintended consequences, including potential effects on cancer risk, immune function, epigenetic regulation, and neurodevelopment, though findings remain conflicting, and causality is not established. These controversies have motivated interest in 5-MTHF as an alternative fortificant and indicate the need to better understand how competition between folic acid and 5-MTHF at FRα might influence tissue folate delivery in vulnerable populations [32–34].

**Fig. 1:**
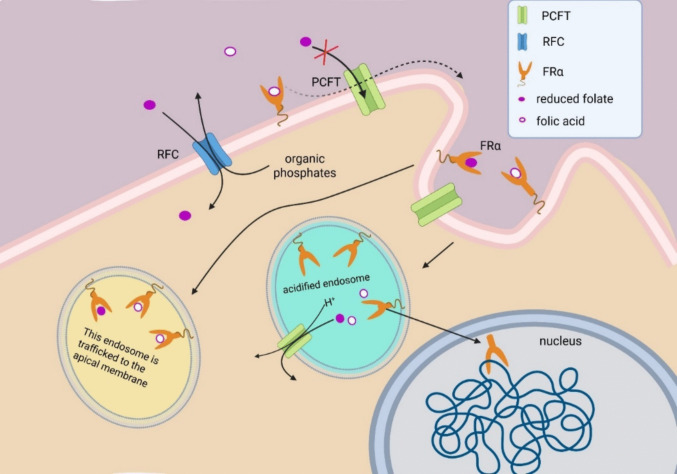
Folate transport into the cell. The following proteins are known to mediate folate passage across the plasma membrane: reduced folate carrier (RFC, SLC19A1), proton-coupled folate transporter (PCFT, SLC46A1), and folate receptors (FRs). Of these, only RFC and FRs mediate folate entry into the cell under conditions relevant to the blood-brain barrier. RFC functions as an antiporter; it facilitates folate uptake in exchange for intracellular organic phosphate. Folate receptors, represented here by FRα, are internalized by endocytosis and therefore do not directly release folate into the cytosol. Instead, PCFT, being co-internalized with FRα, enables folate transport into the cytosol following endosomal acidification [38, 39]. After internalization of the FRα-folate complex in endosomes and endosome acidification, FRα can be cleaved from its GPI anchor and transported to the nucleus, where it binds to specific chromatin sites. Nuclear translocation appears to be regulated by post-internalization-dependent lysine acetylation and serine phosphorylation on FRα. Another possible scenario is intracellular trafficking of FRα-folate-containing endosomes through the cytosol, followed by exocytosis of exosomes carrying FRα-folate complex, a process proposed to occur in choroid plexus [40]

Although membrane localization of FRα has mainly been studied in malignant cells, which may limit generalizability, it is believed to be concentrated in *lipid rafts*—cholesterol- and sphingomyelin-rich microdomains of the cell membrane that are more structured than surrounding regions [35, 36]. Folate-bonded FRαs are internalized by endocytosis and may undergo two different future directions. One possibility is that, after endosome acidification, folate is dissociated from FRα and is transported into cytosol through PCFT; FRα can be cleaved from its GPI anchor and transported to the nucleus (Fig. 1), where it binds to AT-rich chromatin regions [18, 37]. Nuclear translocation appears to be regulated by post-internalization–dependent lysine acetylation and serine phosphorylation of FRα. Modified forms of FRαs lacking certain lysine or serine residues are able to translocate to the nucleus even in the absence of folate binding [15].

Another possibility observed in the choroid plexus is that endosomes are transported through the cytosol to apical regions of the plasma membrane, where FRα–folate complexes are secreted in association with exosomes [18, 40] (Fig. 1).

**Fig. 2:**
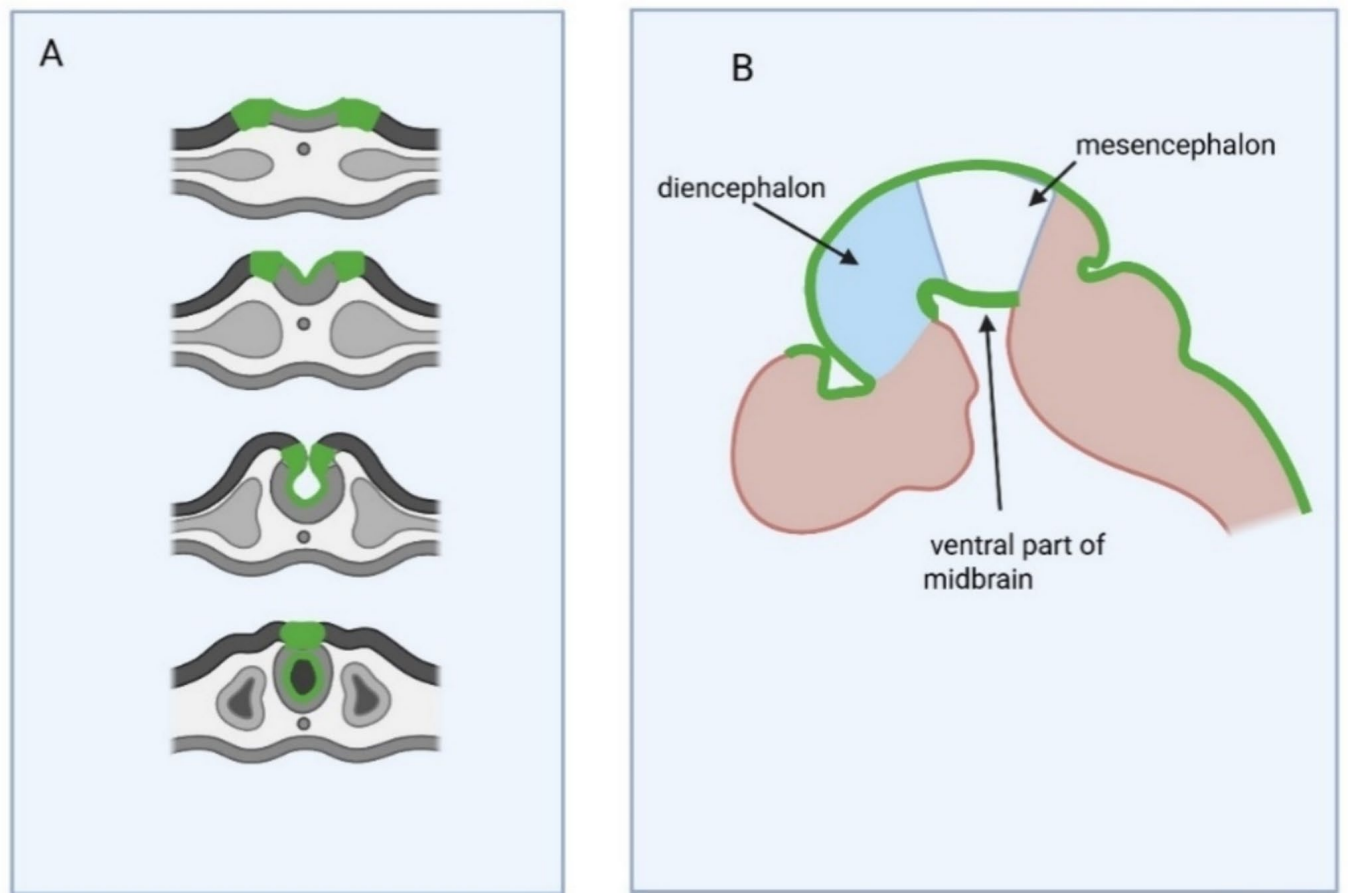
The scheme of FRα expression in the developing murine nervous system. **A** FRα expression during neurulation. **B** FRα expression in forebrain after neurulation. FRα expression patterns are shown in green. Expression first appears on the dorsal edges of the neural plate, near the region where the neural plate folding begins. The expression patterns then expand toward the folding spreading, preceding the folding process

Expression patterns of FRα in mammalian nervous system development were explored on mice embryos. During neurulation, the first stage of CNS development, FRα is highly expressed in apical parts of the neural plate [41] (Fig. 2). Expression first appears on the dorsal edges of the neuroectoderm above the notochord, near the region where the neural plate folding begins. The expression patterns then expand toward the folding spreading, preceding the folding process. Later, from embryonic day 10.5, FRα expression also spreads to the ventral parts of the diencephalon [41]*.*

Expression patterns during human CNS development are not described; high FRβ and low FRα expression are reported in human fetal brain [24]. The method of measurement does not allow the authors to distinguish expression localization, and the timing of expression is not defined either. However, FRα expression at the early developmental stages of vertebrate models, such as *Xenopus laevis* and chicken embryos [14, 42], supports the suggestion that FRα expression during neurulation is evolutionarily conserved.

**Fig. 3:**
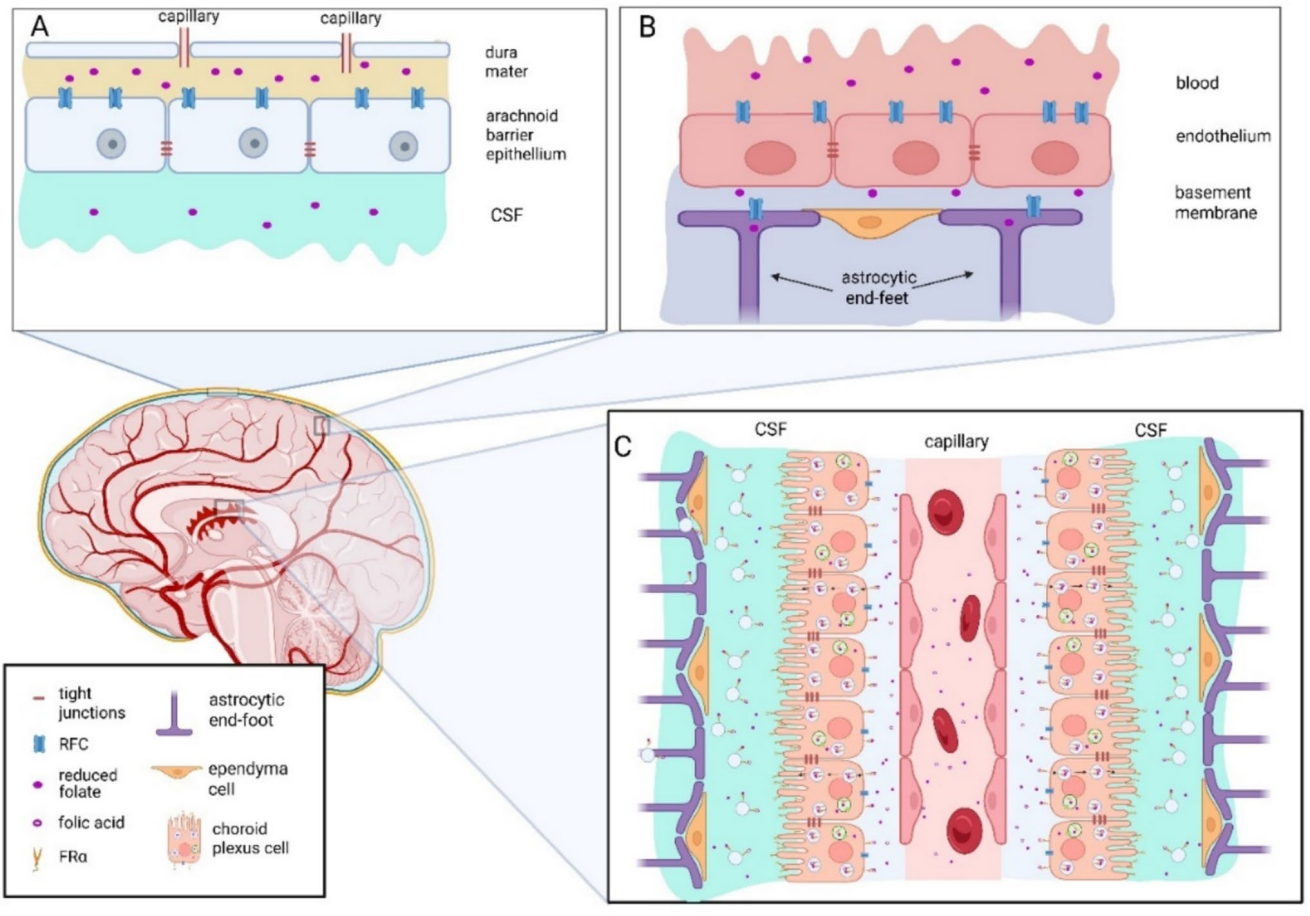
Folate transport into brain parenchyma through arachnoid barrier, blood-brain barrier and choroid plexus. (A) Arachnoid capillaries (arachnoid barrier). (B) Parenchymal capillaries (blood-brain barrier). (C) Choroid plexus. Folate is essential for the development and proper functioning of the CNS. Under normal physiological conditions, folate concentration in human blood serum is less than half the concentration found in cerebrospinal fluid (CSF). Importantly, sufficient folate levels in CSF can be maintained even when serum folate is low [47], and conversely, CSF folate deficiency can occur despite normal serum folate concentrations. Folate is delivered to the CNS through several pathways: arachnoid capillaries (arachnoid barrier), parenchymal capillaries (blood-brain barrier), and the choroid plexus. Current understanding suggests that folate transport across the blood-brain and arachnoid barriers relies on the reduced folate carrier (RFC) (A and B), which has a lower affinity for folate than folate receptor alpha (FRα), especially for the oxidized folate forms as folic acid. In contrast, FRα is exclusively expressed on neuroepithelial cells of the choroid plexus (C), playing a key role in maintaining CNS folate levels, as commonly measured by CSF 5-MTHF concentrations

In the adult murine and human CNS, FRα is found to be abundantly expressed in the choroid plexus [9, 24] (Fig. 3), which where it is involved in transporting folate to other compartments [38, 40]. Although histological studies have not revealed expression of FRα in brain parenchyma [9], there is indirect evidence for transient FRα expression in the hippocampus of adult mice [16] and in peripheral neurons [11]. The expression appears to be dynamically regulated by extracellular folate concentration [43, 44], activation of estrogen receptors [45], or by agents activating the mTOR signaling complex [46]. FRα expression has also been detected in cultured neuronal progenitors and differentiated neurons [19]. These facts suggest that FRα expression can be re-established under specific conditions.

## Role of FRα in Nervous System Development and Function

In mouse embryos, FRα expression initially appears along the dorsal edges of the neural plate, at the sites where folding begins, and consistently precedes neural plate folding [1] (Fig. 2). Expression in the neural plate during vertebrate neurulation was supported in another research [14, 21]. Later, from embryonic day 10.5, FRα expression also emerges in the ventral parts of the diencephalon, mesencephalon, and the optic vesicle. By embryonic day 15.5, FRα expression becomes concentrated in the choroid plexus and along the margins of the retina. In the adult brain, FRα expression remains present in the choroid plexus (Fig. 3) but has not been detected by immunohistochemical methods in the brain parenchyma [9].

Interestingly, FRα expression in the ventral diencephalon and mesencephalon spatially overlaps with regions that give rise to dopaminergic neurons [48]. In vitro studies further demonstrate that dopaminergic progenitors and early dopaminergic (DA) neurons express FRα [19]. These findings suggest that FRα may participate in early dopaminergic lineage specification or maturation, although its precise functional role in vivo remains to be clarified.

Experimental modulation of FRα receptor activity demonstrates that proper FRα signaling is essential for neural tube closure in vertebrate models. Mouse embryos lacking FRα fail to undergo neurulation and are resorbed, even in the presence of sufficient folate [17]. Amphibian embryos are not dependent on external folate for development; however, successful neurulation still requires FRα activation by either folate or metabolically inactive agonists. Similarly, formation of neural tube-like rosettes in human neural progenitor cell cultures depends on functional FRα [20], as does neurulation in zebrafish embryos [49]. These findings show that FRα is essential for the neurulation process not only due to its transport function but also through structural or signaling roles.

One aspect of its structural function is supporting cell polarity in the neural plate, which is critical for primary neurulation. Proper cell polarity relies on adherens junctions connecting the apicolateral membranes of adjacent cells [50]. When FRαs cannot bind ligands, neural plate cell polarization is disrupted, even in embryos with normal folate levels. Conversely, internalization of FRαs, even when bound to metabolically inactive ligand, restores normal neurulation. FRα activation supports cell polarity by downregulating endocytosis as well as C-cadherin ubiquitination and degradation, thereby promoting the formation of stable adherens junctions in the neural plate [20].

Another function of FRα is modulating calcium (Ca^2^⁺) signaling [20]. Although the precise mechanism remains unclear, evidence from *C. elegans* indicates that FRαs modulate Ca^2^⁺-permeable ion channels of transient receptor potential superfamily, melastatin-related subfamily (TRP/TRPM) upon binding to folate [51–54]. In vertebrates, TRP/TRPM channels are essential for successful neurulation [55], which makes them plausible mediators of FRα–folate-induced Ca^2^⁺ transients in these systems as well. Additionally, studies in mouse and chicken embryos suggest that folate receptors may also interact with other membrane-bound proteins, such as Src family tyrosine kinase Lyn [56] and NMDA receptor [57]. However, the exact mechanism linking FRα to these membrane proteins during neurulation remains unclear and warrants further investigation.

During development, FRα overactivation may partially compensate for impaired activity of other proteins, thereby preventing embryonic lethality. For instance, a loss-of-function mutation in Shroom3 typically causes 100% incidence of neural tube defects in mouse embryos. Short-term supplementation with folic acid, but not 5-MTHF, reduces this incidence to 72% and improves embryo survival [58]. This selective effect of folic acid is consistent with FRα involvement, given its higher affinity for folic acid compared to other folate forms. Mechanistically, Shroom3 regulates cytoskeleton organization by coordinating Rho and Rho-associated kinase (ROCK). ROCK activity, in turn, activates myosin II, drives actomyosin constriction, and reduces the apical surface of neuroblasts. The interaction between Shroom3 and ROCK is essential for proper neurulation and acts in concert with WNT/planar cell polarity signaling [59, 60]. Ligand-bound FRα can also induce actomyosin constriction via alternative kinases such as myosin light chain kinase and proto-oncogene tyrosine-protein kinase Src [61], potentially compensating for insufficient Shroom3 activity by providing an alternative pathway to support apical constriction. Similarly, FRα activation helps mouse embryos overcome Paired Box Gene 3 (Pax3) deficiency [62, 63]. In vitro studies further show that, upon activation, FRα translocates to the nucleus and binds promoters of Pax3 downstream genes [12, 64] (Fig. 1). In this way, FRα may regulate cell-fate determination by binding to promoters of regulatory genes such as Pax3 or Notch, and by decreasing histone H3K27 methylation. Indeed, FRα nuclear internalization increases the expression of genes associated with the pluripotent neuronal state, including those involved in transcriptional regulation, growth factor signaling, G-protein activity, and cell proliferation. In contrast, the absence of FRα activity leads to upregulation in cell apoptosis markers and downregulation of genes typically associated with pluripotent cells [65–70], suggesting a role for FRα in extending the undifferentiated state of neuroblasts. However, the exact mechanisms by which FRα activation corrects developmental processes remain to be experimentally elucidated.

In summary, FRα activation has broad effects on molecular processes at the cell membrane, in the cytosol, and in the nucleus. It can modulate cation channels activity [20, 52], adherens junction density, endocytosis rates [20], and enzyme phosphorylation [56, 61] and act as a transcription factor with the capability to activate compensatory pathways [12, 64]. These pathways may enable embryos to overcome defects in regulatory proteins and help maintain neuroblasts in an undifferentiated state.

## FRα Expression and Function in the Adult Brain

In the adult brain, FRα remains abundant in the choroid plexus. Histological studies report FRα being primarily localized on the apical membrane of choroid plexus epithelial cells, with lower levels on their basolateral membrane and some presence in endosomal compartments within the cytoplasm [9, 38, 40]. Choroid plexus is a highly vascularized structure located in all brain ventricles. It consists of a single layer of neuroepithelial cells surrounding a dense network of capillaries. FRα has been found on both the apical and basolateral membranes of these neuroepithelial cells, as well as within intracellular vesicles [9, 38]. Folate transport through the choroid plexus occurs mainly via endocytosis of FRα-bound folate. Two potential pathways follow [40] (Fig. 3).

- Acidification of the endosomes leads to folate dissociation from FRα, followed by transport into the cytoplasm via proton-coupled folate transporter (PCFT), supporting intracellular folate storage.
- Endosomes are transported to the apical side of the cell, and the FRα–folate complex is secreted into the CSF via exosomes.

Thus, CNS may receive folate in two forms: free folate and folate bound to FRα in exosomes. If confirmed, it would be important to determine whether these different folate pools serve distinct biological roles.

Even if histological methods fail to reveal the presence of FRα in brain parenchyma, numerous studies provide both direct and indirect evidence for FRα expression and function in differentiated neurons. Mechanistically, FRα-containing exosomes from CSF are internalized by astrocytes and can be transported to neurons [40]. However, after direct injection of folic acid or FRα-binding peptide into the hippocampus of aged mice, FRαs were detected within neuronal nuclei [16], demonstrating that FRα can be expressed in differentiated neurons themselves. Other studies show that tau protein can upregulate neuronal FRα expression [71], and that folic acid supplementation enhances hippocampal neurogenesis, modulating Notch signaling in the neurons [72]. Because folic acid is primarily transported via FRα, these results support the possibility that adult neurons express FRα in a context-dependent manner.

Outside CNS, FRα expression has been directly observed in axonal growth cones during axonal regeneration. The expression is required for successful regeneration [11]. FRα supports axonal growth through changes in DNA methylation, potentially regulating the expression of *Gadd45a*, which is associated with the control of neurite outgrowth [73, 74]. Recent studies on aged animals suggest that FR*α* activation can also shift neuronal gene expression patterns toward a more juvenile profile [16]. The shift is accompanied by decreased extracellular matrix density typical of the young brain and enhanced neuronal plasticity in dentate gyrus cells. These findings appear to be consistent with FRα’s ability to maintain cells in an undifferentiated state during development.

As mentioned earlier, FRα was found on DA progenitors during CNS development; however, DA neurogenesis has also been reported in adult mice [75]. It remains unclear whether DA progenitors in adult brain also express FRα or whether they acquire different properties. The evidence indicates that FRα activity is required to support neuron progenitors in the adult brain [16, 37], which allows us to suggest that even DA progenitors require FRα expression and activity throughout development and adulthood. Thus, FRα may be transiently expressed in various types of neurons, under conditions involving repair, regeneration, and neuroplasticity. Such short-lived and low-intensity expression may fall below the detection threshold of conventional immunohistochemical methods, particularly when compared to choroid plexus, where FRα levels are extremely high. The functional significance of FRα in differentiated neurons warrants further investigation.

## FRα-Mediated Signaling Pathways in Glial Differentiation and Survival

In glial cells, FRα likely plays a role similar to that observed in neurons, promoting differentiation during development. Indeed, FRα activation in the developing CNS can promote astrocyte differentiation via the JAK-STAT (Janus Kinase–Signal Transducer and Activator of Transcription) pathway [65, 66]. A folic acid supplementation study suggests that FRα activation may also be important for oligodendrocyte survival and proper myelination during CNS development, since folic acid is transported mainly through this high-affinity receptor [67]. In that study, folic acid increased the expression of genes involved in folate metabolism, cytoskeleton organization, transcriptional regulation, and myelin formation. However, the specific role of FRα in oligodendrocyte differentiation and survival has not yet been directly confirmed and still needs experimental verification.

FRα activation may also promote a shift in glial cells toward a less differentiated state, similar to its effect on mature neurons. The dedifferentiation of glial cells depends on FRα’s ability, upon activation, to translocate into the nucleus and modulate histone methylation and gene expression [15]. In aged animals, folic acid deficiency exacerbates the pro-inflammatory microglia response in a model of ischemic brain injury [68], thereby contributing to increased neuronal damage under folate-deficient conditions. Although that study did not directly assess FRα, the use of folic acid for the nondeficient group suggests the possibility of FRα-mediated effects, indicating that FRα activation may exert anti-inflammatory action in the adult brain and could be beneficial.

In summary, FRα serves both as a regulator of folate delivery and transcription factor in glial cells, impacting cell maturation, plasticity, and injury response. Elucidating these pathways modulated by FRα, including its role in nuclear signaling, chromatin remodeling, and histone methylation, could be an important frontier for research into neurodevelopmental conditions.

## Clinical Implications of FRα in Neurodevelopmental and Neuropsychiatric Conditions

FRα activity is vital for multiple stages of human CNS development: from neurulation, including neural tube closure through myelination and circuit refinement and into adulthood where it supports synaptic plasticity, neuroplasticity and cognitive function [13, 14, 16, 24, 69, 70, 76]. By sustaining brain folate pools, FRα supports nucleotide synthesis, one-carbon (C1) metabolism, epigenetic methylation, and redox homeostasis critical to neuronal and glial functioning. In addition, FRα can act as a gene expression regulator, influencing neural and glial cell fate, plasticity, and cognitive functions [14–16, 18, 65, 77]. Disruption of FRα-mediated transport, therefore, provides a mechanistic link between systemic folate biology and a spectrum of neurodevelopmental and neuropsychiatric conditions [22, 70, 78–80].

Variants in folate pathway genes such as MTHFR 677C>T or FOLH1 can reduce methylation capacity or folate bioavailability, particularly in the context of low dietary folate, but typically do not by themselves explain profound CNS folate depletion [81–87]. However, impaired FRα activity often leads to neurological dysfunction even when peripheral folate concentrations remain within normal levels. This phenomenon is exemplified by cerebral folate deficiency (CFD) [22], a neurometabolic disorder characterized by low cerebrospinal fluid 5-MTHF despite normal serum levels. CFD typically arises from genetic mutations in the FRα gene or from the presence of circulating autoantibodies directed against FRα (FRAAs) [22, 24, 87–89]. Thus, FRAAs represent a major clinically relevant mechanism linking folate dysregulation to neurodevelopmental and neuropsychiatric conditions including autism spectrum disorder (ASD) [21, 90], treatment-resistant schizophrenia [91], and depression [22, 92].

Blocking FRAAs (bFRAAs) interfere with folate binding and receptor-mediated endocytosis, lowering CSF 5-MTHF despite adequate systemic folate and leading to a “functional” brain-specific folate deficiency. Maternal FRAAs during pregnancy have been associated with increased risk of neural tube defects and adverse neurodevelopmental outcomes in animal models, suggesting that in utero FRα blockade may perturb early patterning and later circuit formation [69, 70, 93]. Moreover, these antibodies are highly prevalent in neurodevelopmental conditions, such as ASD, and detected in approximately 60–75% of affected children [90]. The association between maternal bFRAAs and neurodevelopmental conditions has been shown in animal models [77] but, to our knowledge, has not yet been reported in clinical practice.

Mechanistically, impaired FRα-mediated folate uptake disrupts C1 metabolism, reducing methylation capacity and altering the expression of genes critical for synaptic formation, neuronal excitability, and neurotransmitter metabolism. These effects collectively contribute to abnormal neurodevelopmental trajectories and cognitive or behavioral dysfunction observed in affected individuals. Moreover, chronic exposure to FRα autoantibodies may elicit inflammation in the choroid plexus, further impairing folate transport and brain homeostasis [21]. FRAAs are thought to arise through dietary exposure to cow milk; thus, people with prolonged exposure to a dairy-rich diet may be at higher risk of developing these antibodies [89, 94].The clinical importance of identifying FRα-related dysfunction lies in its therapeutic potential. Several studies report that supplementation with folinic acid (leucovorin), a reduced and bioavailable folate form that bypasses FRα-mediated transport, can partially restore CNS folate levels and improve neurobehavioral symptoms. In FRAA-positive children with ASD, leucovorin treatment has produced measurable gains in language, attention, and social communication. However, much of the existing clinical evidence comes from a relatively small number of research groups, and methodological variability, particularly differences in FRAA assay sensitivity, limits the comparability of findings across studies [95]. It is also important to consider that excessive folic acid supplementation may have unintended biological effects. Experimental and epidemiological studies suggest that high levels of unmetabolized folic acid in circulation could alter folate receptor expression, transporter dynamics, or downstream methylation pathways, although direct evidence in ASD remains limited [33, 96, 97]. Some data indicate that folate receptor expression may be subject to feedback regulation depending on folate availability [97, 98], but whether supraphysiological supplementation modifies FRα expression or function in the developing brain is not yet fully understood. Larger, methodologically consistent, and population-diverse investigations are therefore needed to clarify both the therapeutic window and long-term safety of folate-related interventions, as well as to support standardization of FRAA detection methods.

In summary, deficient or immune-compromised FRα function provides a key link between metabolic, genetic, and immune contributors to brain disorders. It highlights a subset of patients in whom targeted correction of CNS folate transport via folinic acid or future FRα-directed strategies may modify neurodevelopmental trajectories or symptoms. Moving forward, larger, multi-center studies with standardized FRAA testing, rigorous CSF/serum folate phenotyping, and long-term neurobehavioral follow-up are essential to define who benefits, when intervention is most effective (e.g., prenatal vs early childhood vs adult), and how FRα-centric mechanisms intersect with broader risk architectures in ASD, schizophrenia, and related conditions.

## Conclusion

FRα is the most efficient transporter of folate into CNS, showing high affinity for multiple folate forms. It is crucial for neural tube closure during development and later contributes to neuronal and glial differentiation. Beyond folate uptake, FRα influences membrane dynamics, ion channels, cytoskeleton, and gene regulation. In adults, FRα remains abundant in the choroid plexus, with potential re-expression in neurons and glia suggesting roles in neurogenesis and plasticity. As anti-FRα autoantibodies may be triggered by a dairy-rich diet, common in Western countries, developing reliable methods to detect and assess their functional impact is an important future direction.

## Data Availability

No datasets were generated or analysed during the current study.

## Abbreviations

ASD: Autism spectrum disorder
C1: One-carbon
CFD: Cerebral folate deficiency
CNS: Central nervous system
DA: Dopaminergic
FOLH1: Folate hydrolase 1
FRα: Folate receptor α
FRβ: Folate receptor β
FRγ: Folate receptor γ
FRδ: Folate receptor δ
FRAAs: Folate receptor α autoantibodies
bFRAAs: Blocking autoantibodies to folate receptor α
GPI: Glycosylphosphatidylinositol
MTHFR: Methylenetetrahydrofolate reductase
5-MTHF: 5-Methyltetrahydrofolate
NMDA: N-methyl-D-aspartate
Pax3: Paired box 3
PCFT: Proton-coupled folate transporter
RFC: Reduced folate carrier
SCF: Cerebrospinal fluid
TRP/TRPM: Transient receptor potential superfamily, melastatin-related subfamily

## Declarations

### Figures Source

All figures were created with BioRender by Egorova, O. (2026) https://BioRender.com/trxl2hy.
